# Multisector global collaboration to advance the inclusion of pregnant and lactating people in HIV prevention research

**DOI:** 10.1097/QAD.0000000000004257

**Published:** 2025-08-28

**Authors:** Robin Schaefer, Breanne Lievense, Suzanne Day, Logan Donaldson, Linda-Gail Bekker, Audrey Chigome, Grace Kumwenda, Lisa Noguchi, Chukwunomso Osakwe, Veronica Miller, Anne Drapkin Lyerly, Manju Chatani-Gada

**Affiliations:** a Forum for Collaborative Research, UC Berkeley School of Public Health, Washington; b AVAC, New York, New York; c The University of North Carolina at Chapel Hill, Chapel Hill, North Carolina, USA; d The Desmond Tutu HIV Centre, University of Cape Town, Cape Town; e South African Health Products Regulatory Authority, Pretoria, South Africa; f Jhpiego, Johns Hopkins University, Baltimore, Maryland; g Independent Global Health Advocate, Longmeadow, Massachusetts, USA.

**Keywords:** collaboration, HIV prevention, inclusive research, pregnancy, lactation

## Introduction

In many countries, notably in Africa, pregnancy and lactation are characterized by increased HIV acquisition risks [1,2] due to biological, psychosocial, behavioral, and socio-cultural factors [2–4]. HIV acquisition during pregnancy and lactation contributed to the 350 000 infections among girls aged 15+ and accounted for one-quarter of vertical HIV infections in the African region in 2023 [5]. Therefore, there is an urgent need for access to HIV prevention options during pregnancy and lactation.

Antiretroviral drugs (ARVs) as preexposure prophylaxis (PrEP) are critical for ending the HIV epidemic globally. The WHO recommends tenofovir disoproxil fumarate (TDF)-containing oral PrEP, the dapivirine vaginal ring (DVR), and long-acting injectable cabotegravir (CAB-LA). Additionally, six-monthly injections with lenacapavir (LEN) have recently been found efficacious and well tolerated in phase 3 clinical trials [6]. WHO recommendations cover all people at substantial HIV risk, including pregnant or lactating people, and WHO released guidance on PrEP implementation during pregnancy and lactation [7]. Nevertheless, PrEP programs for these populations remain limited [8], and various factors limit PrEP uptake and use among pregnant and lactating people, including side effects, stigma, and healthcare-related barriers [9].

Pregnancy and lactation have been common exclusion criteria in clinical studies of new drugs, resulting in inadequate safety data. When drugs receive regulatory approval, labels may include limited information on use during pregnancy and lactation [10]. For ARVs, there are often multiple years between initial regulatory approval and pregnancy safety data becoming available [11,12]. Even where regulatory approvals do not restrict use during pregnancy and lactation, national guidelines may recommend against this. These factors pose substantial challenges to implementing novel HIV prevention strategies for pregnant and lactating people.

There are signs of a shift toward increased inclusion of pregnant and lactating participants in HIV prevention research. Although pregnant and lactating people were initially excluded from large clinical trials of the DVR and CAB-LA, subsequent open-label and, in the case of the DVR, additional randomized studies allowed pregnant and lactating participants to receive these products, generating important safety evidence [13–17]. Moreover, in a phase 3 trial on LEN among cisgender women, participants had the choice to use contraception and continue receiving study drugs after becoming pregnant [6].

A symposium at the 5th HIV Research for Prevention Conference (Lima, Peru, October 6–10, 2024), co-hosted by AVAC, the Forum for Collaborative Research (Forum), and PREPARE (PRomoting Equity for Pregnant Adolescents in REsearch), highlighted recent progress with the inclusion of pregnant and lactating people in HIV prevention research [18]. The symposium provided examples of collaborative initiatives to underscore that progress toward more inclusive HIV prevention research is fostered by concerted action by diverse actors working in collaboration, including advocates and community representatives, researchers, international organizations, industry, and regulatory authorities [19–23]. In this article, we describe these initiatives and lessons learned on implementing successful collaborations and argue that multisectoral collaboration is central for advancing more inclusive HIV and global health research.

## Different collaborative approaches, common goals

The symposium highlighted three initiatives around HIV prevention during pregnancy and lactation. AVAC is a global nonprofit that drives ethical and equitable HIV prevention with partners across the field. To advance research including pregnant and lactating people, AVAC, through the Coalition to Accelerate and Support Prevention Research (CASPR) and the PHASES Project, co-led a Think Tank in April 2022 with researchers, industry, community representatives, advocates, regulators, and funders [24]. Informed by key informant interviews and consultations (including with former HIV prevention trial participants) and a review of existing consensus documents, the discussions identified four critical actions. These included centering pregnant and lactating people in research using a reproductive justice lens; ensuring early and meaningful engagement of all relevant parties in trial design and conduct; aligning regulatory frameworks to support pregnancy and lactation-specific data collection; and strengthening ethical review processes to promote responsible inclusion of pregnant and lactating people in HIV prevention research. Key to the findings was the importance of partnering with community representatives and organizations to drive advocacy, ensuring that research efforts reflect the needs and priorities of the affected communities. An action agenda was developed and is being used to track multisectoral efforts in advancing these goals. At the symposium, AVAC advocated for moving beyond siloed approaches and towards more coordinated action to accelerate change, making it clear that advocates and communities are essential to driving progress and must be recognized as equal partners in shaping and implementing these efforts.

The Forum, a public-private partnership at the University of California, Berkeley, has formed a working group, funded by the Gates Foundation, to facilitate knowledge exchange between regulatory authorities and other partners in the USA, Europe, and Africa on PrEP use during pregnancy and lactation. Representatives from regulatory agencies, international organizations, academia, industry, and affected communities met eight times in 2024 to generate consensus on critical data needs on PrEP use during pregnancy and lactation, considering clinical research and postmarketing surveillance. Throughout, the working group emphasized the benefits of cross-regional, cross-sector, and interdisciplinary discussions. At the symposium, the group was represented by a member of the South African Health Products Regulatory Authority who highlighted the implications of excluding pregnant and lactating people in research and the importance of collaborative postauthorization surveillance. Surveillance is needed to gather essential data on longer-term and less common outcomes, particularly related to new products, which clinical trials may not be able to generate [25].

The US National Institutes of Health (NIH)-funded and University of North Carolina (UNC) at Chapel Hill-based PREPARE Project aims to develop guidance for the ethical inclusion of pregnant adolescents in biomedical HIV and co-infections research. PREPARE builds on the findings from the PHASES Project, which developed ethics guidance for inclusion of pregnant adults in HIV/co-infections research that supports research in pregnancy as ethically imperative [19]. PHASES surfaced a need for specific guidance for adolescents. In addition to conducting empirical research with community members, implementing partners, and adolescents who had experienced pregnancy in the USA, Botswana, and Malawi, PREPARE trained and convened Youth Advisory Boards to robustly include youth voices in the development of ethics guidance. In 2024, PREPARE convened an expert working group representing disciplines such as bioethics, pediatrics, women's health, youth advocacy, law, research regulation, and industry. Cross-disciplinary dialogue and youth representation have contributed to the development of novel, concrete, and actionable recommendations for the ethical inclusion of pregnant adolescents in biomedical HIV research. At the symposium, PREPARE shared approaches to cross-disciplinary engagement and the necessity of working collaboratively to develop ethics guidance.

## Multisectoral dialogue and collaboration: A global health asset

AVAC/CASPR, the Forum's working group, and PREPARE demonstrate that collaboration across disciplines, sectors, and regions is feasible, beneficial, and urgently needed to advance equitable and inclusive HIV prevention research. Several key takeaways resulted from cross-project dialogue and symposium discussions, providing insights into how to successfully implement complex collaborations. First, each initiative represents a network of actors, including researchers, ethicists, regulatory authorities, communities, and industry, offering space for dialogue and consensus-building. Overcoming divides between actors is needed to drive change. The organizations facilitating the networks (AVAC, the Forum, and UNC) were not considered “leaders” of the networks; rather, they ensured that activities were implemented efficiently while underscoring the democratic nature of the networks, with everyone having an equal voice. This promoted trust between the parties involved. Second, emphasizing a common goal from the beginning – making well tolerated and effective HIV prevention products accessible to all who could benefit from them – was critical. Third, broad inclusivity was essential to the success of these initiatives, involving a wide range of relevant parties from different regions. Particularly, inclusion of representatives from affected communities and community and national advocates was important to ensure that efforts center on the needs of those who could benefit from research. Finally, the collaborative efforts of these three initiatives resulted in key outputs necessary for advancing inclusive research, including action agendas, knowledge exchange platforms, ethics guidance, and consensus papers. Emphasizing, from the outset, the aim to produce such collaborative outputs helped to focus activities, and developing these outputs was a critical part of the consensus-building process, ensuring buy-in from all parties and accountability.

Establishing collaboration, integrating diverse perspectives and priorities, and, where possible, finding consensus can be lengthy, tedious, and expensive, involving many convenings, incremental progress, synthesis, and compromise. However, these efforts should be seen as an asset, not a burden. There is a growing movement to decolonize global health [26], which will likely only accelerate given recent shifts in the global funding and support for biomedical research and global health programs. This significant shake-up of the global health and funding landscape underscores that communication, cooperation, and collaboration across regions, disciplines, and sectors are needed more than ever to generate ethical, impactful, and efficient research. True collaboration requires equal footing between collaborators, overcoming power imbalances and differences, but also creating benefits and value for everyone. Progress with the inclusion of pregnant and lactating people in HIV prevention research illustrates this, and lessons from the initiatives described in this article can be applied more widely for the advancement of equitable and inclusive global health research.

## Acknowledgements

R.S. drafted the first version of the article and finalized the manuscript with input from all authors. All authors contributed significantly to the discussions underlying this article, including at the scientific symposium at the 2024 HIVR4P conference. The authors thank all participants at the scientific symposium for their engagement and contributions to the discussion.

This work was supported, in whole or in part, by the Gates Foundation [INV-045445]. Under the grant conditions of the Foundation, a Creative Commons Attribution 4.0 Generic License has already been assigned to the Author Accepted Manuscript version that might arise from this submission.

This work was supported by the Coalition to Accelerate and Support Prevention Research (CASPR), made possible by the generous support of the American people through the US President's Emergency Plan for AIDS Relief (PEPFAR) and the US Agency for International Development (USAID). The contents do not necessarily reflect the views of PEPFAR, USAID or the United States Government.

A.D.L. and S.D. are supported by a grant from the National Institutes of Allergy and Infectious Disease of the National Institutes of Health (R01AI108368). The views expressed here are the authors’ and do not necessarily represent those of the National Institutes of Health.

M.C.-G. acknowledges that portions of this work were initiated during her tenure as Director of Partnerships & Capacity Strengthening at AVAC.

### Conflicts of interest

The Forum for Collaborative Research receives unrestricted grants from the pharmaceutical industry, including from companies involved in the development of antiretroviral drugs (ViiV Healthcare, Merck, and Gilead Sciences). These grants are provided to the organization and are not specifically linked to the present work (which is funded through a grant by the Gates Foundation).
